# The Surgical Complications and Sequelæ of Typhoid Fever

**Published:** 1898-12

**Authors:** 


### Book Reviews.

---

THE SURGICAL COMPLICATIONS AND SEQUELÆ OF TYPHOID FEVER. By WILLIAM W. KEEN, M. D., LL. D., Professor of the Principles of Surgery and of Clinical Surgery, Jefferson Medical College, Philadelphia, etc., etc. Five plates and nine engravings. Philadelphia: W. B. Saunders. 1898.

A few months ago in a review of a text-book dealing with general surgery, published in the JOURNAL, it was suggested that future surgical writings should be confined to monographs dealing with one phase of a special subject. No better illustration of this idea could be furnished than by this volume of Dr. Keen's, which is exhaustive and perfect and invaluable to the general practitioner, as well as to the surgeon, dealing as it does with a matter which concerns them both, and which forms a part of their every-day practice. Typhoid fever, experience has shown, is as much within the province of the surgeon as the general practitioner, for while the latter very properly deals with the expression of the fever itself, to the former belongs the after-effects; the gangrene, the joint and bone affections, the abscesses, the ear and glandular troubles, the intestinal perforations and the specific mixed infections.

This work sheds a brilliant light on various phases of typhoid and brings out into sharp relief many things in connection with this very common infection which are too often overlooked; if not overlooked, then too often slighted by the general practitioner, who makes it no part of his business to wield the knife.

The Spanish war with its attendant crop of camp complaints, chief of which, owing to incompetency—not of the Surgeon General or his medical purveyors, but of regimental commanders of volunteer organisations—is typhoid fever, is a most excellent and vivid illustration of the work and its object. The surgical complications and sequels of typhoid have been overlooked long enough, and the work is particularly valuable at this time when soldier wrecks of typhoid fever are wabbling about in every corner of the land, veterans of a war, and victims of their own carelessness and the inefficiency of their immediate superiors.

The volume, so far as literary merit is concerned, is almost beyond criticism. It is seldom one finds in a scientific work so much that is really able and brilliant from a literary view-point. Each division is taken up and carried out to a conclusion with masterly grace and pleasing pen picturing.

The work is based upon tables of 1,700 cases compiled by the author and Dr. Thompson S. Westcott, instructor in diseases of children, University of Pennsylvania, and most carefully has this work been done. The field has been gleaned and not a straw has escaped the gleaners. Incorporated in the book is a chapter on the ocular complications of typhoid fever, by Dr. George E. de Schweinitz, of Philadelphia. As an appendix is given the Toner lecture, No. V., delivered by Dr. Keen in 1876, on the Surgical complications and sequelæ of the continued fevers. An idea of how large a rôle the complications and sequels of typhoid play is shown in the introduction, wherein it is stated that of 2,000 fatal cases tabulated, 24 per cent. died of typhoid infection *per se* and 76 per cent. from the various medical and surgical complications and sequels.

The pathology of the surgical complications and sequels is taken up in the opening chapters, the typhoid bacilli and its viability, inside and outside the body, is described ; as is their wide diffusion in various organs and tissues of the body. Then mixed infections are considered, the pyogenic faculty of the bacilli is proved, and this is followed by a description of typhoid infection of different organs without typical typhoid lesions in the intestines. This is a most important section of the early days, dealing as it does with matters which are largely surgical. Then comes the chapters which deal distinctly with the sequels and complications. Gangrene being one of the earliest sequels is first considered. This condition obtains between the fourteenth and forty-ninth days, most commonly in the second and third weeks. Embolism and thrombosis are minutely considered, and the whole is illustrated with splendid plates in half-tone and numerous clinical reports and descriptions.

Under the general heading of typhoid affection of the joints are considered, rheumatic and septic typhoid arthritis and typhoid arthritis proper and dislocations of the hip-joint. The latter phase is given considerable space, and proof of joint infection is given in a few of the cases cited ; but Dr. Keen is by no means satisfied with the findings and expresses the hope that the near future will bring forth definite conclusions regarding the rôle of the typhoid bacillus in joint affections. Typhoid affections of the bones and typhoid abscesses are thoroughly discussed, as are the hematomata and the cerebral complications, the latter being illustrated by half-tone reproductions of microscopical sections. Glandular and laryngeal affections and the typhoid affections of pleura, lungs and heart and of the esophagus and stomach are valuable studies, including as they do complete reports of cases illustrative of the widespread pyogenic power of the typhoid bacillus.

Special reference should be made to the chapter on intestinal perforation, which is a masterpiece. Beginning with a brief historical sketch of surgical interference it considers the frequency of perforation and the statement is made that in children it is rare; that it is more frequent in men than in women. The greatest number of perforations occurred in patients between twenty and thirty years of age; the largest number occurred in the third week. Among many other points of importance and interest considered are: duration of life after perforation and the importance of the presence or absence of leucocytosis. The dangers of surgical interference are considered calmly and impassionately. Statistics and half-tone illustrations complete a most valuable addition to surgical literature.

Liver, gall-bladder and spleen are taken up and their typhoid affections minutely described and carefully considered, and Dr. Keen's portion of the work concludes with a chapter on the specific mixed infections, which is up to the general high standard of the entire work.

Dr. de Schweinitz's chapter on the ocular complications is exhaustive and is in itself a most valuable contribution to medical literature. Every phase of the matter in hand is touched upon, even to the medico-legal point regarding the responsibility of a general practitioner and his liability for failure to call in a specialist in the event of eye complications arising.

The final chapter of conclusions is one of the most carefully prepared and interesting sections of the whole book.

The Toner lecture is too well known to need more than a passing reference. Everything in the work has been brought right up to the time of going to press, and in every chapter one finds after a general consideration, a description of pathology, symptoms, cause, duration and treatment of the complication in question. Certainly would American surgical literature be enriched if all writers were to follow Dr. Keen's example and confine themselves to brainy monographs, leaving the literary hash of general text-books to the hacks who manufacture public school books on scientific subjects.

